# Toward Systems Biomarkers of Response to Immune Checkpoint Blockers

**DOI:** 10.3389/fonc.2020.01027

**Published:** 2020-06-24

**Authors:** Óscar Lapuente-Santana, Federica Eduati

**Affiliations:** ^1^Department of Biomedical Engineering, Eindhoven University of Technology, Eindhoven, Netherlands; ^2^Institute for Complex Molecular Systems, Eindhoven University of Technology, Eindhoven, Netherlands

**Keywords:** tumor microenvironment, precision immuno-oncology, multi-omics profiling, systems biology, predictive biomarkers, cancer signaling networks, immune checkpoint blockers

## Abstract

Immunotherapy with checkpoint blockers (ICBs), aimed at unleashing the immune response toward tumor cells, has shown a great improvement in overall patient survival compared to standard therapy, but only in a subset of patients. While a number of recent studies have significantly improved our understanding of mechanisms playing an important role in the tumor microenvironment (TME), we still have an incomplete view of how the TME works as a whole. This hampers our ability to effectively predict the large heterogeneity of patients' response to ICBs. Systems approaches could overcome this limitation by adopting a holistic perspective to analyze the complexity of tumors. In this Mini Review, we focus on how an integrative view of the increasingly available multi-omics experimental data and computational approaches enables the definition of new systems-based predictive biomarkers. In particular, we will focus on three facets of the TME toward the definition of new systems biomarkers. First, we will review how different types of immune cells influence the efficacy of ICBs, not only in terms of their quantification, but also considering their localization and functional state. Second, we will focus on how different cells in the TME interact, analyzing how inter- and intra-cellular networks play an important role in shaping the immune response and are responsible for resistance to immunotherapy. Finally, we will describe the potential of looking at these networks as dynamic systems and how mathematical models can be used to study the rewiring of the complex interactions taking place in the TME.

## A Change in the Landscape of Biomarkers Discovery

Tumor cells are able to activate several mechanisms to evade the immune response by disguising themselves as “self” cells. Binding to inhibitory checkpoint molecules (i.e., immune checkpoints) they can block antitumor activities of the immune system. Immunotherapy with immune checkpoint blockers (ICBs) uses antibodies to target immune checkpoints, such as PD1, PD-L1, and CTLA-4, unleashing the immune response. In clinical trials, ICB therapy has been shown to achieve durable therapeutic response and to increase patient survival in different cancer types, although still a small number of ICBs are FDA-approved (1, 2). Even if clinically approved, ICB therapy is effective for a small subset of patients. Given the potential immunological toxicity (3, 4) and the elevated costs (>US$100,000 per patient per year) (5) associated with ICBs, it is of paramount importance to be able to predict which patients will likely respond to the therapy, in order to administer the optimal treatment based on biomarkers.

**Figure 1:**
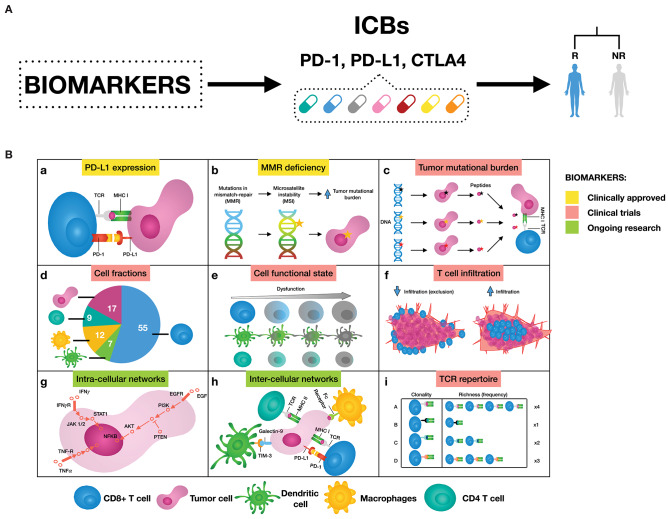
**(A)** Biomarkers can help to select patients that are likely to respond to immune checkpoint blockers (ICBs), leaning toward personalized immuno-oncology. **(B)** Examples of possible mechanisms that can inform on response to ICBs. (a) Binding of PD-L1 to PD-1 transmits an inhibitory signal that reduces the proliferation of T cells and can induce apoptosis. (b) Genetic alterations in MMR proteins produce a large number of mutations leading to a large number of mutations that generate neoantigens. (c) The number of non-synonymous single nucleotide variants in a tumor, known as the tumor mutational load, may affect the generation of neoantigens influencing the T cell response. (d) Quantification of different immune cell types offers a new opportunity to assess treatment efficacy. (e) Immune cells can be in a dysfunctional state, therefore promoting tumor immune escape. (f) The degree of T cell infiltration can affect ICBs effectiveness. (g) Signaling pathways are responsible for the correct functioning of the cells. Cancer is caused by deregulations in these cellular signaling pathways, ultimately changing the cell phenotype. (h) There are a great number of pathways and cross-talks involved in the communication between tumor and immune cells. (i) Both, all sorts of TCR sequences and the richness of each specific TCR sequence, can deal with the wide variety of neoantigens expressed by tumor cells. As a result, more specific T cell clones are present, and therefore ready to mount an effective T cell response.

The investigation of mechanisms supporting immune resistance has provided a great opportunity for biomarker discovery of patient response to ICBs (Figure 1). Two biomarkers have been clinically approved for PD-1/PD-L1 blockade therapy: the first is immunohistochemistry (IHC) staining of PD-L1 in non-small-cell lung cancer (NSCLC), melanoma, renal cell carcinoma (RCC), urothelial cancer, and triple-negative breast cancer (TNBC) (6); and the second is high microsatellite instability/defective mismatch repair (MSI-H/dMMR) regardless of tumor type (7, 8). Other emerging predictive biomarkers such as tumor mutational burden (TMB) (9, 10), signatures of a T cell inflamed tumor microenvironment (TME) either alone (10) or in combination (11), and neoantigen load (12–14) are still undergoing clinical trials. In addition, T cell receptor (TCR) diversity has been used as a biomarker to monitor the clonal expansion of T cells in breast cancer, glioma, cervical cancer, and leukemia/lymphoma (15–18). Further efforts both to exploit the utility of these biomarkers and to search for additional ones are still ongoing. For a complete review of these biomarkers and in which tumors they work, we refer to Havel et al. (19).

Despite being promising, these biomarkers also present some limitations. For instance, IHC enables measuring PD-L1 expressed on tumor cells, however the expression of this biomarker fluctuates over time and varies between different tumor sites. This variability undermines the ability to evaluate PD-1/PD-L1 therapies effectiveness based on IHC, as reviewed in Topalian et al. (20) and Camidge et al. (21). Another example is TMB, which is known to correlate imperfectly with clinical response (12, 13, 22). Neoantigen burden should partially overcome this issue, however most computational tools fail to estimate true neoantigens (19, 20, 23), and additional features should be considered to better determine neoantigen immunogenicity as reviewed in Finotello et al. (24).

Above-mentioned examples shed light upon the conceptual problem of looking only at individual components of the TME. While the characterization of different parts playing a role in the interaction between tumor and immune system has been essential to elucidate the most important actionable mechanisms, further research is required to define biomarkers harnessing a more coordinated joint action of these mechanisms. Predictive biomarkers for immunotherapy with ICBs have been extensively reviewed previously (19, 20, 23, 25). In this Mini Review we focus on how a holistic profiling of the TME can provide new opportunities for identifying systems-based biomarkers built on existing synergies between the different individual components of the TME. Such a shift toward multifaceted strategies has been favored by increasingly available multi-omics data from bulk populations, individual cells, and imaging technologies (26), that can be integrated using computational approaches. In the following sections we will describe how biomarkers can be derived by considering three increasing levels of complexity. The first is the cellular component, focusing on the immune contexture of tumors, such as immune cells quantification, functionality, and localization. The second is the network of communication between and within cells of the TME. Finally, we will elaborate on how mathematical models can be used to take the dynamic nature of these networks into account.

## The Role of the Immune Contexture on ICB Efficacy

It is well-known that different types of immune cells can play a different role in the response to ICBs (27). For example, while the presence of CD8+ T cells within the TME is a good biomarker of ICBs efficacy, a high abundance of regulatory T (Treg) cells is generally associated with poor prognosis. Different tools have been developed to quantify tumor-infiltrating immune cells from bulk (RNA-seq) and single-cell (scRNA-seq) RNA sequencing measurements, as extensively reviewed in Finotello and Eduati (26) and Finotello and Trajanoski (28).

Apart from quantification of immune cells, their spatial localization also plays a pivotal role in the response to immunotherapy (29). For instance, CD8+ T cells not only need to be present, but also to be infiltrated (hot tumor) for the ICB therapy to work. In fact, pure quantification of CD8+ T cells is not always associated with favorable prognosis (30). Imaging techniques can be used to explore the spatial patterns of immune infiltration. A notable example of a biomarker assessing through IHC, both the abundance and the location (tumor center and invasive margin) of two lymphocyte populations (CD3+ and CD8+ T cells) is the immunoscore (31), that was shown to accurately predict patient survival in colorectal cancer patients (32). More recently, spatial information of CD8+ T cells from IHC was integrated with transcriptomics data to study the effect of lymphocyte infiltration in patients with TNBC, providing predictive biomarkers of ICBs response (33). Automatic approaches for image analysis could reveal useful in the future for high-throughput identification of spatial biomarkers. A first attempt in this direction was the development of tumor infiltrating lymphocytes maps by using deep learning on images from the cancer genome atlas (TCGA) (34).

Another important factor that affects patients' response to ICBs is the functional state of the different immune cells (35). Dysfunctional states of T cells can be characterized from bulk and single-cell RNA-seq (36–38) and epigenetic profiling (39–41). ICBs aim at rescuing dysfunctional T cells, therefore the investigation of their functional state can inform on ICBs therapy success and limitations (36–39, 41). Depending on the type of stimulatory signal, macrophages (42, 43), and B cells (44, 45) can develop into functional subsets that have either positive or negative effects on tumors. Another example are dendritic cells (DCs), that normally control cancer antigen presentation, priming and activation of T cell responses, however the TME can compromise their ability to stimulate the immune response (46, 47). Certain computational tools for cell-type quantification can also unmask the phenotypic state of cell subpopulations in the TME by inferring the transcriptomics profiles of individual cells (48, 49). A promising research direction for biomarkers discovery is also given by new technologies that allow generation of omics data from tissue slides preserving cell spatial identity (50, 51). These approaches would result in combined localization and characterization of the cells in the TME.

Analysis on the immune infiltrate quantification, functionality, and localization can help both to explain the diversity of the tumor immune milieu and develop informative biomarkers for ICBs (27, 52, 53). Pointing in this direction, different efforts have recently explored the use of bulk transcriptomics data to derive more complex immune-related scores to assess the likelihood of a patient to respond to ICBs (38, 54–63).

## Intra- and Inter-Cellular Networks Orchestrate the Immune Response

The functional state of cells in the TME is defined by a complex system of communication between molecules within the cells (intra-cellular networks) and among different cells (inter-cellular networks). Looking at intra- and inter-cellular networks can provide a more holistic perspective of the TME and inform a new class of biomarkers for immunotherapy and its potential combination with other targeted therapies (64).

**Figure 2:**
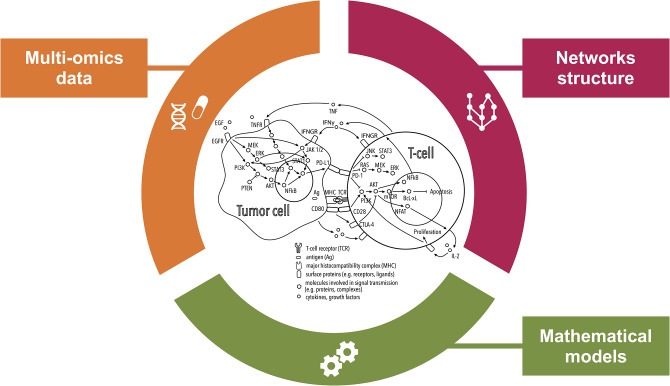
Comprehensive inspection of the tumor microenvironment (TME) from multiple angles. This illustration shows how systems medicine approaches are key to improving our understanding of the mechanisms of resistance to immunotherapies with checkpoint blockers (ICBs). Combining multi-omics data and prior knowledge information on inter- and intra-cellular pathways using mathematical models allows encompassing the complexity of the TME paving the way for the discovery of systems-based biomarkers of the response to ICBs.

Intra-cellular signaling pathways play a part in shaping the interaction with the immune system [(65, 66); Figure 2]. Abnormalities in tumor-intrinsic signaling, involving oncogenes and tumor suppressor genes, have been associated with mechanisms of inherent immune resistance (67). Examples are PTEN loss (68) or EGFR gain of function (69), both causing PI3K-Akt pathway activation and leading to over-expression of PD-L1 and consequent immunoresistance. Due to the complexity of signaling pathways, with numerous cross-talks and feedback loops, the adoption of individual oncogenic drivers as biomarkers is not expected to be effective in most cases (20). In fact, PD-L1 signal is directly regulated by numerous oncogenic pathways such as Ras, mTOR, EGFR, MEK, ERK, and MAPK (70). Besides pathways regulating immune checkpoints, other signaling cross-talks control the immune response from different perspectives, like inactivation of TP53 or activation of β-catenin pathway, both reducing chemokine production by tumor cells and thereby reducing recruitment of immune cells into the TME (71, 72).

In addition, cancer cells receive signals from other cells in the TME through ligand-receptor interactions. These inter-cellular communications lead to changes in the phenotype of the regulated cells thus playing an important role in both progression and prognosis of cancer (73, 74). An example is the response elicited on cancer cells by two cytokines (TNF and IFNγ) produced by activated T cells. These cytokines induce PD-L1 expression through JAK-STAT and NF-kB signaling, inducting acquired resistance to the immune response (75, 76). Another study identified a relationship between high expression of NOS2 and prolonged IFN signaling in tumors resistant to PD-1 blockade (77).

While collections of intra- (78) and inter-cellular (79) interactions can be derived from literature and databases, additional data are required to characterize the networks for each patient or group of patients. Transcriptomics and proteomics data can provide the basis to study intra- and inter-cellular signaling networks. Imaging data can also be integrated to improve our understanding on spatial localization of interacting cells. Computational methods have been developed to infer integrated inter- and intra-cellular networks from bulk (80, 81) and single-cell (81, 82) RNA-seq data. These tools could be exploited to derive biomarkers for immunotherapy by studying the functional effect of cell-cell communication. In a recent study, a curated database of ligand-receptor interactions (79) was integrated with gene expression data to deconvolute the transcriptional profile of cancer and stromal cells and infer cross-talks in the TME (83). Interestingly, the authors show that for different cancer types, PD-L1 expression is higher on cancer or stromal cells which nicely correlates with the general responsiveness to immunotherapy. Further research is required to assess if this holds also for individual patients, making it potentially a more effective biomarker than bulk PD-L1 expression. In another recent publication (84), researchers performed an extensive literature curation to derive a comprehensive signaling network of innate immune response in cancer, including cell type-specific signaling in macrophages, DCs, myeloid-derived suppressor cells, and natural killer cells. Such network was then integrated with scRNAseq data from macrophages and natural killer cells in melanoma to study the heterogeneity of innate immune cell types and could potentially be used to predict patient survival and response to immunotherapies. Finally, Worzfeld et al. combined parallel bulk transcriptomics and proteomics data on tumor cell spheroids, tumor-associated T cells and macrophages to derive inter-cellular signaling networks in the ovarian cancer microenvironment (85). Such networks included several immune checkpoint regulators and appeared to have potential clinical relevance. Overall, these studies have demonstrated the enormous benefit that holistic approaches combining complex multicellular networks can bring into the immuno-oncology field, and we expect that in the forthcoming future more research efforts will be spent in this direction. The recent developments of 3D cell culture models resembling the TME, are expected to be a powerful tool for further *in vitro* and *ex vivo* investigation of intra-cellular communication, and to study their effect on the response to ICBs (86).

## The Potential of Looking at the Dynamicity and Plasticity of the TME

It is well-known that the cellular functional state changes dynamically in response to environmental changes and perturbations such as drug treatment (87, 88), calling for identification of the dynamic properties of the networks. The ideal data for dynamic functional characterization of the system's response are obtained upon perturbation (89). Functional screening of the effect of cancer drugs has been so far focused on cancer cell lines. While cell lines are a debatable model system, they proved to be a valuable tool to explore novel biomarkers of drug response (90, 91). High-throughput drug screening studies are now also being increasingly performed on organoids (92) or other 3D experimental models (86), which are more physiological human cancer models of the TME. These efforts open new ways for pre-clinical investigation of the effect of immunotherapy. Finally, more recent technologies allow screening also of patient biopsies without need for culturing steps (93–95) paving the way for functional characterization of *ex vivo* tumor samples potentially improving personalized cancer treatment.

To capture the functional context of the immune response, statistical, and mathematical approaches are developing into more compendious methods that integrate multi-omics data and prior knowledge on network structure (Figure 2). While mathematical models do not fall into the standard definition of biomarkers, they can provide predictions of response to immunotherapy. Additionally they can be used to define dynamic biomarkers based on properties of the modeled system, as opposed to static biomarkers that only consider the initial conditions of the system (88).

Dynamic mathematical models can be used to study intra-cellular networks of the different cell types populating the TME (96). To characterize these networks at the patient-specific level, models of signaling pathways in cancer cells have been trained from perturbation experiments (97, 98), gene expression data (99), or integrating multi-omics data (100). The resulting parameters corresponding to these personalized models can be relevant biomarkers of clinical outcome (99–101). Mathematical models have also been used to study intra-cellular signaling in T cells. This includes the investigation of how PD-1 leads to deactivation of the T cell receptor signaling (102) or mechanistic understanding of T cell exhaustion (103). PD-1 is one of the main targets of ICB, and exhausted T cells have a higher number of targetable checkpoint proteins like PD-1 and CTLA-4, therefore the investigation of these aspects could be relevant to identify possible biomarkers.

More studies are now focusing on mathematical models incorporating inter-cellular interactions to better capture the complexity of the TME. Agent-based models can be used to simulate the interactions between cells in the tumor microenvironment seen as a 2D or a 3D grid (104). Each cell is seen as an agent that can perform different tasks with a certain probability (e.g., cells can non-proliferate, divide, or die). Since the immune response can be seen as a probabilistic outcome of a complex system (88), agent-based models are an adequate mathematical approximation to capture this stochasticity. These models can be refined using a multitude of data types and used to simulate the effect of immunotherapy (105, 106), providing a variety of possible outcomes given the same initial conditions that can be interpreted as probability of success. It has been shown that tumor-bearing inbred mice, which have only minimal differences, can respond differently to immunotherapy (88), therefore having models that can incorporate stochasticity provides an interesting approximation of the *in vivo* situation. Another approach to model cell-cell communication is by using response-time modeling (107), where cells are modeled as a black-box that can receive inputs (e.g., cytokines) from other cells, process them, and change state (e.g., immune cells can switch between inactive and active) accordingly with a certain probability. Recently, Grandclaudon et al. combined perturbation data with a multivariate quantitative model to study context dependent interactions between DCs and helper T cells (108). A different approach based on quantitative systems pharmacology was recently used to simulate the effect of ICB therapy in metastatic breast cancer patients using a four compartments (central, peripheral, tumor-draining lymph node, and tumor) model (109).

Additionally, combining mathematical models with longitudinal data, i.e., data collected at different time points, can be used to investigate the evolutionary dynamics of treatment response. This aspect is particularly relevant, especially to be able to distinguish at an early stage real tumor progression (patient should be assigned to a different treatment) from what is called pseudoprogression, i.e., temporary progression followed by a response to the treatment (patient should be kept on ICB). The latter behavior has been described using a model of immune activation incorporating the dynamics of antigen presentation (110). Based on a system of three ordinary differential equations to describe the interaction between tumor cells, Treg cells, and cytotoxic T cells, this model could explain why, in response to ICBs, the tumor can worsen before starting regressing. Other multi-cellular models have been used to derive *in silico* patients to test different possible dynamics of treatment response (111, 112), that could be compared with longitudinal measurements of tumor load from PET/CT imaging (112). Longitudinal data are often limited to non-invasive imaging and, in a few cases, to transcriptomics, IHC, TCR, and genome sequencing data (113, 114) for a limited number of time points due to invasiveness of biopsies. Computational modeling of longitudinal data is still at its infancy, but we envision that in the future more mechanistic dynamic models will be able to exploit this type of data for definition of dynamic biomarkers.

## Conclusions and Future Perspectives

Current limitations in identifying predictive biomarkers for ICB therapy are partially due to overlooking the complexity of the TME. Following the advancements in technologies to measure multi-omics data, measurements of bulk populations, individual cells, and spatial information have paved the way to a more comprehensive view of the TME. Recent efforts are focused on searching for signatures of response to ICBs that consider quantification, localization, and functionality of different immune cells in the TME, showing improved predictive power with respect to simpler biomarkers (115). However, they still miss an integrative strategy that takes a view of the whole TME, rather than examining each factor in isolation. In this respect, mechanistic models incorporating existing biological basis, e.g., on intra- and inter-cellular pathways, can accompany both therapy and biomarker development in immuno-oncology (116).

There is compelling evidence that the interplay of the immune system, tumors, organs, and external environment, harmonizes antitumor immune responses (117). Therefore, we envision that novel systems medicine approaches entailing mathematical models can gradually build up a profile of the TME, both in the lab and, more importantly, in the clinic. To this end, building patient specific models have become of increasing importance, especially when based on data that can be measured in clinical settings. Moreover, systems approaches can especially be useful to provide rationale for alternative personalized treatments such as combinatorial therapy.

## Author Contributions

ÓL-S and FE wrote and edited the article. All authors contributed to the article and approved the submitted version.

## Conflict of Interest

The authors declare that the research was conducted in the absence of any commercial or financial relationships that could be construed as a potential conflict of interest.

## Abbreviations

CTLA-4: cytotoxic T lymphocyte antigen 4
DC: dendritic cell
ICB: immune checkpoint blocker
IFNγ: interferon gamma
IHC: immunohistochemistry
MMR: DNA mismatch repair
MSI-H: high microsatellite instability
NOS2: nitric oxide synthase 2
NSCLC: non-small-cell lung cancer
PD-L1: programmed cell death-ligand 1
PD-1: programmed cell death protein 1
RCC: renal cell carcinoma
RNA-seq: RNA sequencing
scRNA-seq: single-cell RNA sequencing
TCGA: the cancer genome atlas
TCR: T cell receptor
TMB: tumor mutational burden
TME: tumor microenvironment
TNBC: triple-negative breast cancer
TNF: tumor necrosis factor
Treg: regulatory T cell.
